# Mutations of CX46/CX50 and Cataract Development

**DOI:** 10.3389/fmolb.2022.842399

**Published:** 2022-02-11

**Authors:** Yumeng Shi, Xinbo Li, Jin Yang

**Affiliations:** ^1^ Key Laboratory of Visual Impairment and Restoration of Shanghai, Department of Ophthalmology and Visual Science, Eye Ear Nose and Throat Hospital of Fudan University, Shanghai, China; ^2^ Casey Eye Institute, Oregon Health and Science University, Portland, OR, United States

**Keywords:** gap junction, Cx46, Cx50, cataract, lens microcirculation, oxidative stress

## Abstract

Cataract is a common disease in the aging population. Gap junction has been considered a central component in maintaining homeostasis for preventing cataract formation. Gap junction channels consist of connexin proteins with more than 20 members. Three genes including GJA1, GJA3, and GJA8, that encode protein Cx43 (connexin43), Cx46 (connexin46), and Cx50 (connexin50), respectively, have been identified in human and rodent lens. Cx46 together with Cx50 have been detected in lens fiber cells with high expression, whereas Cx43 is mainly expressed in lens epithelial cells. Disrupted expression of the two connexin proteins Cx46 and Cx50 is directly related to the development of severe cataract in human and mice. In this review article, we describe the main role of Cx46 and Cx50 connexin proteins in the lens and the relationship between mutations of Cx46 or Cx50 and hereditary cataracts. Furthermore, the latest progress in the fundamental research of lens connexin and the mechanism of cataract formation caused by lens connexin dysfunction are summarized. Overall, targeting connexin could be a novel approach for the treatment of cataract.

## Introduction

Cataract is the opacity of lens and the most important cause of low vision and blindness worldwide. Cataract can be divided into metabolic cataract, age-related cataract, congenital cataract and others. With the increase of the elderly population, there are more and more aged-related cataract. Congenital cataract is the main cause of blindness in children, exerting a dramatic impact on their quality of life. Therefore, the prevention and treatment of cataract is particularly important. Lens homeostasis is critical to its transparency, and its imbalance can lead to cataract.

The lens is a biconvex transparent tissue situated between the iris and the vitreous, composed of a single layer of epithelial cells under the anterior capsule and the enormous lens fibers differentiated from epithelial cells (Ruan et al., 2020). Epithelial cells at the lens equator region migrate laterally toward the equator, where they transform into differentiating fiber cells and finally turn into mature fiber cells through extensive cell elongation. The lens is able to transmit light via the contraction or relaxation of the ciliary muscle and focus light onto the retina (Summers et al., 2021). In order to increase light transmission and minimize light scattering, various organelles including the Golgi apparatus, endoplasmic reticulum, and nucleus are degraded in the differentiating lens fibroblasts (Brennan et al., 2018; Brennan et al., 2021). In addition, lens crystallins are at high concentration in the lens to enable appropriate refractive ability that aids in light transmission and focusing (Cvekl and Eliscovich, 2021).

**FIGURE 1:**
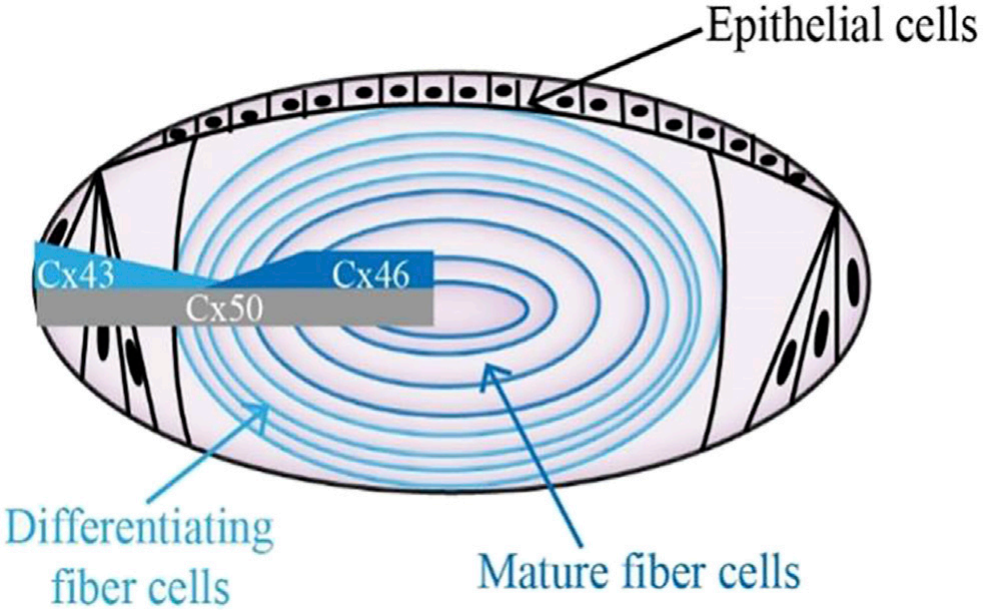
Diagram showing the distribution of connexin hemichannels in the lens. Connexin isoform Cx43 is mainly expressed in the anterior epithelial layer. Cx46 can be found in the differentiating lens fibroblasts and mature lens fibroblasts. The expression of Cx50 stays the course of entire lens development.

Gap junction channels are critical in regulating the lens microcirculation system, which is crucial for the motion of the ions and other medium to maintain lens homeostasis (Brink et al., 2020; Valiunas et al., 2019; Valiunas and White, 2020). Moreover, gap junctional communication is a way to maintain normal lens fiber cells physiology and tissue functions (Van Campenhout et al., 2021). Gap junction channels facilitate these processes by permitting the selective passage of ions and other molecules, forming both electrical and biochemical coupling between cells. Gap junction channels are assembled by the coaxial alignment of two hemichannels. Six connexin molecules oligomerize into a hemichannel (also called connexon) (Beyer and Berthoud, 2014). Connexins are a family of structurally related transmembrane proteins in humans with approximately 20 members. Every single connexin protein consists of four transmembrane domains (T1-T4), two extracellular loops (EL1, EL2) with a cytoplasmic loop (IL), and cytoplasmic N-terminal and C-terminal components (Figueroa et al., 2019; Mese et al., 2007; Sánchez et al., 2019). Three connexins presented in the lens are α1 (Cx43), α3 (Cx46), and α8 (Cx50), which are encoded by three genes: Gja1, Gja3, and Gja8, respectively (Yue et al., 2021; Ping et al., 2021). In the layer of lens epithelial cells, abundant expression of Cx43 could be detected, whereas Cx46 is exclusively present in the lens fiber cell, where its expression corresponds with fiber cell differentiation, and Cx50 is widely expressed in both lens epithelial and fiber cells (Figure 1) (Paul et al., 1991; Delvaeye et al., 2018; Ceroni et al., 2019; Tong et al., 2021). Although the pathogenesis of cataracts is not yet fully clear (Davison, 2020; Hashemi et al., 2020; Shiels and Hejtmancik, 2021; Taylan Sekeroglu and Utine, 2021), a number of studies have shown that disruption of lens connexin hemichannels proteins Cx46 and Cx50 expression are associated with cataract formation (White et al., 1998; Chang et al., 2002; Addison et al., 2006; Xia et al., 2006a).

## CX46 and CX50 in Cataract Formation

### Mutations of Cx46 and Cx50 Identified in Human and Rodents With Cataracts

More than 40 different mutations associated with cataractogenesis have been identified in the gene region of GJA3 and GJA8 in human pedigrees (Table 1). The first variant P-to-S transition at site 88 in Cx50 was identified in a British family with zonular pulverulent or “dust-like” cataracts (Mese et al., 2007). Subsequently, two mutations in the GJA3 gene have also been reported in different families with inherited congenital cataracts (Mackay et al., 1999).

**TABLE 1:** Summary of Cx46 and Cx50 mutants associated with cataract formation.

Mutation	Cataract type	Family origin	References
*Human Cx46*			
N63S	zonular pulverulent	British	Mackay et al. (1999)
P187L	nuclear pulverulent	Chinese	Rees et al. (2000)
R76H	zonular pulverulent	Australian	Ping et al. (2021)
N188T	nuclear pulverulent	Chinese	Li et al. (2004)
V44M	nuclear	Chinese	Chen et al. (2017)
*Human Cx50*			
P88S	zonular pulverulent	British	Mese et al. (2007)
E48K	zonular nuclear pulverulent	Pakistani	Berry et al. (1999)
V64G	nuclear	Chinese	Sharan et al. (2005)
P88S, P88Q	lamellar pulverulent	British	Arora et al. (2006)
T56P	nuclear	Mauritanian	Hadrami et al. (2019)
S217P	perinuclear	Chinese	Li et al. (2019)
*Rat Cx46*			
E42K	nuclear	Cataract rat strain	Yoshida et al. (2005)
*Mouse Cx50*			
A47A	nuclear	No2 cataract mouse	Steele et al. (1998)
V64A	nuclear and zonular cataract and microphthalmia	Mouse Aey5 generated by ENU	Graw et al. (2001)
G22R	microphthalmia and dense cataract	Lop10 mutation cataract mice	Chang et al. (2002)
S50P	whole cataract and small eye	ENU mutagenesis	Xia et al. (2006b)
*Rat Cx50*			
R340W	cataract	UPL rat strain	Yamashita et al. (2002)

More variants of these two connexin hemichannels have been reported in families in recent years. A heterozygous G-to-A substitution in the exon region of GJA3 gene was detected and resulted in the replacement of Asp with Gly at the N-terminus of Cx46 protein in a Chinese family with congenital nuclear pulverulent and posterior polar cataract (Rees et al., 2000). Another Cx46 variant, R76H, was identified in a large Australian cataract pedigree with zonular pulverulent cataract by using linkage analysis (Ping et al., 2021). Most of these mutations in the Cx46 protein are present in the N-terminal, the first transmembrane, and extracellular domains. One missense mutation N188T and another frameshift mutation at the position S380Qfs of Cx46 gene were found to be related to hereditary autosomal dominant cataract in two different Chinese families (Paul et al., 1991; Li et al., 2004). In addition, a missense mutation in the Cx46 coding region occurred in a Chinese cataract pedigree, giving rise to the dysfunction of the Cx46 protein, which might be potentially linked to the development of congenital nuclear cataract. Methionine substituted for valine at site 44 (V44M) in the Cx46 gene is responsible for that mutation (Chen et al., 2017).

Similar to Cx46, numerous mutations of the Cx50 gene have been identified. The first Cx50 mutation from a British family with zonular pulverulent cataract was identified at the second transmembrane domain of the encoded gene (Mese et al., 2007). Subsequently, Glu48Lys was the second recognized mutation reported in a three-generation Pakistani family (Berry et al., 1999). A missense variation V64G of Cx50 conserved residues in a Chinese family occurred at the phylogenetically conserved extracellular loop1 (Sharan et al., 2005). The autosomal dominant lamellar pulverulent cataract from a four-generation British family is associated with two mutations located at P88S and P88Q of GJA8, resulting from a 262C > A transition (Arora et al., 2006). In addition, an insertion mutation at codon 203 of GJA8 was mapped in a southern Indian family with autosomal recessive cataract, producing a functionally null allele and the subsequent reduction of transmembrane domain, cytoplasmic domain, and the second extracellular domain, and was different from the vast majority of mutations recognized with dominant features (Ponnam et al., 2007). Recently, a new variation at site 166 (c.166A > C) in Cx50 coding region was confirmed by the comprehensive screening by next-generation sequencing in a Mauritanian family with congenital nuclear cataracts (Hadrami et al., 2019). Moreover, a novel missense mutation of c.217T > C in a four-generation Chinese family with autosomal dominant congenital cataract (ADCC) was identified, resulting from a serine-to-proline interchange at residue 73 of the Cx50 gene (Li et al., 2019).

In addition to humans, mutations of Cx46 and Cx50 in homozygous mice can cause cataracts. Targeted deletion of GJA3 and GJA8 genes in mice can develop into a dominant or semi-dominant cataract pattern. Abundant mutations have been reported in mice. A single A-to-C transversion within codon 47 was amplified and sequenced in the Cx50 protein-coding regions in No2 cataractous mouse, resulting in congenital hereditary cataracts (Steele et al., 1998). Furthermore, an ethylnitrosourea mutagenesis screen analysis revealed a new cataract mutation, Val-to-Ala interchange at codon 64 of Cx50, in mice with phenotypically hereditary congenital cataracts (Graw et al., 2001). Lens opacity 10 (Lop10) mutation at chromosome 3 and a missense single transversion (G-to-C) in the Cx50 coding region was identified in a mouse that developed microphthalmia with dense cataracts, resulting in Gly-to-Arg substitution at codon 22 (Chang et al., 2002). Moreover, another variant S50P in the Cx50 protein was reported to be associated with smaller lens (Xia et al., 2006b). Apart from mice, connexin mutations have also been detected in rats with cataracts. A C-to-T transversion located at codon 340 in the Cx50 genes was strongly associated with the development of cataracts in the Upjohn Pharmaceuticals Limited (UPL) rat model (Yamashita et al., 2002). A missense mutation at site E42K in the coding region of Cx46 from rats with congenital nuclear cataracts was reported (Yoshida et al., 2005). Only a few mutations in rodents have been utilized for the investigation of gap junction channel, and therefore it is necessary for us to broaden the related studies.

### The Relationship Between Connexin Hemichannels and Cataract Formation

Mathia et al. pointed out that the lens develops an internal circulation system that deliver water, ions, and solute for lens cells to replenish its lack of blood supply (Mathias et al., 2007). It allows nutrients and ions to enter the lens from both the anterior and posterior and to migrate to the center across the extracellular spaces, and unwanted metabolites to exit at the lens equator. The lens is full of plentiful and functional ion channels and transporters that support the internal circulation system. Dysfunction of the lens circulation system has been postulated to linked to cataract formation (Berthoud et al., 2020). Lens gap junctions formed by two oligomeric subunits referred to as hemichannels (also called connexons) display a critical effect on the lens internal circulation system. Both Cx46 and Cx50 form functional homomeric/homotypic gap junction channels and hemichannels. *In vitro* studies demonstrate that majority of lens connexin mutations linked to congenital cataracts will decrease coupling conductance and influence lens circulation (Gong et al., 2007; Berthoud and Ngezahayo, 2017). Most mutations of the Cx46 and Cx50 gene leading to cataracts are recognized as autosomal dominant, but several mutations that have been investigated are non-functional in terms of expression systems (Gerido et al., 2003). Apart from that, connexin variants with increased hemichannel activity could affect lens circulation through cell depolarization, which would reduce the ability of ions and other signals to migrate throughout the organ.

As previously reported, Cx50 knockout mice developed smaller eyes and lens—32 and 46% size reduction in the mass of control littermates, respectively (Gerido et al., 2003). Several studies observed that targeted deletion of GJA8 in mice led to delay in cell denucleation, indicating an important part of Cx50 in lens fibroblast maturation and epithelial cell proliferation (Graw et al., 2001; Sellitto et al., 2004). The expression of Cx50 in place of Cx46 by gene knock-in did not rescue epithelial proliferation, implying that Cx50, but not Cx46, facilitates normal lens growth and development after growth factor stimulation (Yamashita et al., 2002; White et al., 2007; Minogue et al., 2017).

Substantial studies have revealed that knockout of Cx46 gene in mice leads to the impairment of lens transparency and the development of nuclear cataracts, probably caused by accumulation of crystallin cleavage products and production of an insoluble complex of disulfide-associated polypeptides (Gong et al., 1997). In addition, the coupling conductance was completely eliminated when the lens fiber matured, while the conductance in differentiated fibers was greatly reduced. Cx46 deletion-induced nuclear cataracts are also strongly correlated with the elevation of intracellular Ca^2+^ and corresponding change of increased protein degradation in lens fiber cells (Baruch et al., 2001). Change in gap junction communication due to mutations in the lens may be one of the important reasons for the formation of cataracts (Sharan et al., 2005; Schadzek et al., 2019).

Recent studies demonstrated that mutations in connexin hemichannels have a great impact on the function of gap junction channels. A missense mutation with an Asp-to-Ala substitution at site 47 in the first extracellular domain of Cx50 protein in No2 mice resulted in the loss of ability to produce functional gap junction channels, leading to cataractogenesis (Katai et al., 1999). A G-to-A transition mutation at position 139 was identified in the coding region of Cx50 from a family with autosomal dominant nuclear pulverulent cataracts, and also resulted in the loss of ability to generate functional gap junction channels in paired oocytes (Schadzek et al., 2019). Mixed hemichannels consisting of normal and abnormal Cx50 or Cx46 proteins in the lens displayed remarkably altered gating properties and coupling conductance, which may give rise to cataract formation. It is still unknown what the specific role of connexin hemichannels in the lens is.

## Possible Mechanisms of Cataracts Related to Lens Connexin

### Lens Microcirculation and Biomineralization

It is generally known that gap junction channels could maintain the homeostasis of ocular lens by propagating lens microcirculation. Under normal conditions, the circuit of the lens microcirculation is completed when Na^+^/K^+^-ATPase or Na^+^/Ca^2+^ exchanger and Ca^2+^-ATPase on epithelial cells transport Na^+^ and Ca^2+^ ions out of the lens when these intracellular ions are located at the surface of cell (Delamere and Tamiya, 2004; De Maria et al., 2018). Such pumps can produce low intracellular sodium and calcium concentration and form an electrochemical environment (Alvarez et al., 2001; Alvarez et al., 2003; Okafor et al., 2003). To maintain the Na^+^/Ca^2+^ gradient, gap junction channels of the lens regulate circulation system through passive diffusion. Disruption of the lens microcirculation has been implicated in cataract pathogenesis. In the normal mouse lens, differentiating fiber gap junctions facilitate sodium ion flow to the equator once it enters the intercellular compartment. However, it has been found that the intercellular concentration of Na^+^ becomes promoted in lenses isolated from mice expressing Cx46-and Cx50-dominant mutants (Gao et al., 2018). Moreover, loss of Cx46 causes calcium accumulation and subsequent elevation in the activity of Lp82, which is a type of Ca^2+^-dependent protease that generate γ-crystallin cleavage products (Baruch et al., 2001; Ebihara et al., 2003). Measurement of calcium in Cx46 knockout has demonstrated that loss of intracellular coupling leads to the blockage of the efflux path to accumulate Ca^2+^ (Gao et al., 2004). There is also a hypothesis that reduction of Cx46 and Cx50 levels alter the function of gap junction channels in regulating the circulation of lens internal mediums, bringing about further changes to other major components in the lens microcirculation. These experimental evidences offer additional support that calcium displays different distribution patterns in wild-type, knockout and knock-in lens in microcirculation models.

Calcium has also been reported to be tightly related to the development of cataracts (Gerido et al., 2003). Different etiologies of cataract lenses in humans and mice contained increased Ca^2+^ (Vanden Abeele et al., 2006). Elevation of intracellular calcium concentration and corresponding elevated protein degradation in lens fibroblasts due to loss of Cx46 gene are associated with nuclear cataract formation (Liu et al., 2015). Calpain II, a kind of Ca^2+^-dependent protease, induces the development of nuclear cataracts in Cx46 knockout lenses by cleaving crystallin proteins (Baruch et al., 2001). Proteolysis caused by calpain has also been shown to play a role in the truncation of Cx50 (Xia et al., 2006a). Gap junction coupling is also impaired due to sharply declined levels of Cx46 and Cx50 proteins and elevated total calcium concentration in cataract lens from homozygous β-crystallin S11R-mutant mice (Li et al., 2010). Abundant investigations demonstrate an important role of calmodulin (CaM) in maintaining functional gap junction channels. Increased Cx hemichannel activity is mediated by increased intercellular Ca^2+^ concentration and the activation of CaM. The voltage from oocytes expressing Cx46 G143R loses control of hemichannels, which forms a leaky gate, leading to diminish voltage-dependent ionic conductance (Li et al., 2008). A sequence of results showed that loss of cell-cell communication impairs the movement of ions such as Na^+^ and Ca^+^ towards the epithelium, inducing an alteration of [Na^+^]_i_ and [Ca^+^]_i_ gradient in Cx46fs380 mice lenses (Berthoud et al., 2014). These alterations lead to a vicious spiral that could ultimately exacerbate the occurrence of cataracts. Thus, extrapolation to humans shows that people suffering from severely declined levels of connexin or damaged gap junction function may develop cataracts on account of lens microcirculation disorders.

Numerous observations suggest that accumulation of insoluble calcium salts results in the development of cataracts. It probably likely that Ca^+^ would precipitate due to the high concentration of more than 1 μM in the center of the lens, forming insoluble calcium salts (Berthoud et al., 2019). Moreover, using Alizarin acid staining identified immobile and insoluble Ca^+^ in cataractous lenses from Cx46 and Cx50 knockout mice (Gao et al., 2018). These finding may be consistent with calcium oxalate or calcium carbonate crystals found in cataracts patients.

Biomineralization occurs when insoluble precipitates comprising inorganic ions deposit and form mixed particles. Impaired lens circulation in Cx46 and Cx50 knockout mice caused cataracts though Ca^+^ accumulation, precipitation, and biomineralization (Gao et al., 2013). Moreover, modification of the connexins, including via proteolysis, ubiquitination, and phosphorylation, may alter lens microcirculation and affect subsequent biomineralization in the lens (Retamal et al., 2019). The mixed deposits in cataractous lenses might comprise of aggregated non-functional proteins and precipitated Ca^+^. Detection of the Ca^+^ values in cataractous human lenses revealed that the insoluble lens fraction contained a higher proportion of Ca^+^ than the soluble part. Lens biomineralization is probably the main reason for the development of cataracts of additional pathogenesis.

### Age-dependent Truncations

It is universally acknowledged that age-related connexin modification could deteriorate the intercellular communications between lens cells. Over 90% of downregulated expression of Cx46 and Cx50 proteins has been detected in normal lens fiber cells isolated from a group of cataracts patients aged more than 50 years old (Gong et al., 2021). The expression of Cx46 and Cx50 proteins displayed age-dependent reduction, whereas Cx43 remained relatively stable in aging mice. Two mutations in the Cx46 and Cx50 code region, Cx46V139M and Cx50V275I, respectively, were identified with mild association with the development of age-related cataracts in a Chinese population (Zhou et al., 2011). These mutants show the impact on alterations in post-translational modifications (PTMs) of connexin proteins because of age of appearance of cataracts. Polymorphisms in the intronic region of the Cx50 gene and a C-to-G substitution in the code region of Cx46 gene might be linked to the formation of age-related cataracts (Liu et al., 2011; Zhou et al., 2011). Previous studies indicate that an age-dependent decrease of gap junction conductance induces alterations in the ability of ion channels and related transporters in the lens. There is a hypothesis that elimination of over 65% of connexin proteins caused by age-related modifications is responsible for the declined coupling levels in the lens.

With increasing age, truncations in the cytoplasmic loop region and N-terminal domain of Cx46 and Cx50 accumulate in the core, resulting in decreased coupling conductance (White et al., 2007). In addition, the corresponding abundance of these truncations was remarkably altered with aging of lens fiber cells, showing the highest level of truncation products in the nucleus of the oldest fiber cells and the lowest level in the outer cortex of younger, differentiating fiber cells. Previous studies in rodent lens indicated that the levels of age-related connexin hemichannel truncations in younger lenses were lower than those found in older lenses. It is likely that the PTMs of these connexins are dependent on the age of the lens (Rozema and Ní Dhubhghaill, 2020; Fan and Monnier, 2021). The epithelial cells of lens differentiate into fiber cells and the C-terminal of Cx46 and Cx50 proteins are cleaved during this process. The endogenous Cx50 truncations resulted from the enzymolysis of calpain or other proteases. Mass spectrometry analysis identified several truncation sites of Cx46 and Cx50 proteins in bovine lens. C-terminal truncation at site V284 of Cx50 induced nonfunctional hemichannels; in contrast, truncation at position TM4 had no influence on its properties (Slavi et al., 2016). Therefore, cleavage of Cx50 by calpain is able to decrease the proportion of functional connexin hemichannels, and give rise to reduced level of gap junction coupling during lens development. The calpain activity decreases with age in the Cx46 knockout lenses. C-terminal cleavage of Cx46 has no impact on coupling conductance, and ionic permeability of connexin hemichannels composed of truncated Cx46 possessed almost the same function as the full-length isoform (Fan and Monnier, 2021). However, the mechanism attributed to truncations in Cx46 and Cx50 with differentiation and aging remains to be determined.

### Other factors: Oxidative Stress and Hypoxia

Oxidative stress is responsible for the production of highly reactive oxygen species (ROS) and subsequent cellular damage at protein and DNA level has been observed in cataractous lens (Babizhayev and Bozzocosta, 1994; Lin and Takemoto, 2005). To combat constant oxidative stress from the environment, ocular tissue normally produces high concentrations of reduced glutathione (GSH) and utilizes a complicated antioxidant defense system composed of superoxide dismutase (SOD) and glutathione peroxidase (GPX). It is widely recognized that GSH plays an important role in maintaining redox homeostasis and lens transparency (Ho et al., 1997; Delamere and Tamiya, 2004). Depletion of GSH in newborn mice compromise lens transparency and eventually leads to the development of cataract (Laver et al., 1993). Plentiful evidence has been gathered to inform that cataract formation can result from oxidative stress, decreased level of GSH, and the mixed protein-thiol and protein-protein disulfide bonds. Increased levels of GSH and oxidized glutathione (GSSG) have been measured in the core of lens as it ages (Lim et al., 2020). Misfold proteins caused by mutations in some of the connexins presumably deposit in the Golgi bodies or endoplasmic reticulum (ER) to trigger stress responses and ultimately damage crystalline proteins. The Cx46fs380-mutant mice exhibited reduced total levels of β-crystallins consistent with degradation, modification, and truncation of the proteins (Minogue et al., 2005). A decreased GSH level was only observed in the nucleus of homozygous Cx46fs380 lens (Jara et al., 2020). However, a single mutation of P-to-S transversion at amino acid residue 88 of human Cx50 protein resulted in cytosolic aggregates and led to decreased degradation. In addition, a higher level of GSH was observed in homozygous Cx50D47A lens about 2 months old (Jara et al., 2020). Detection of the level of GSH in the lens from connexin-knockout mice suggested that Cx46 (not Cx50) is essential for the movement of GSH from lens cortical cell to lens nuclear cell, under the condition that both Cx46 and Cx50 hemichannels assist in the transport of GSH (Serebryany et al., 2021). Mutation in the Cx46 gene region in mice led to the development of lens opacity and cataracts due to deposit of insoluble polypeptides caused by aggregation of crystallin cleavage products (Gong et al., 1997; King and Lampe, 2005; Kelly et al., 2007).

It has been suggested that targeted deletion of GPX-1 in mice can cause declined expression level of Cx46 and Cx50 together with extremely low level of coupling conductance (Wang et al., 2009). Apart from that, hydrogen peroxide was reported to keep Cx50 hemichannels open, and can assist in the movement of reductant glutathione into lens fiber cells. Both Cx50P88S and Cx50H156N mutations suppress permeability activity of Cx50 hemichannels (Shi et al., 2018). In addition, oxidative stress cause by 4-hydroxynonenal (4-HNE) can deprive the natural properties of Cx46 protein through its carbonylation (Retamal et al., 2020). These mutants ultimately induce apoptosis of lens epithelial cells and fiber cells.

A hypoxic condition is necessary for normal growth and development of the lens. Increased exposure to oxygen has been proven to be a threatening cause for the occurrence of age-related cataracts and nuclear cataracts (Brennan et al., 2020). *In vivo* studies showed that physiological hypoxia is indispensable for inhibiting cell proliferation and preserving smaller lens size (Zhao et al., 2020). Hypoxia might be a critical factor that regulate the expression and function of Cx46 in natural lens. The Cx46 promoter showed tight transcriptional responses when cultured with 1% oxygen in human lens cells (Molina and Takemoto, 2012). Further studies will be needed to elucidate the change of oxygen concentration in responding to the expression of connexin proteins in the lens.

## Conclusion and Future Directions

**FIGURE 2:**
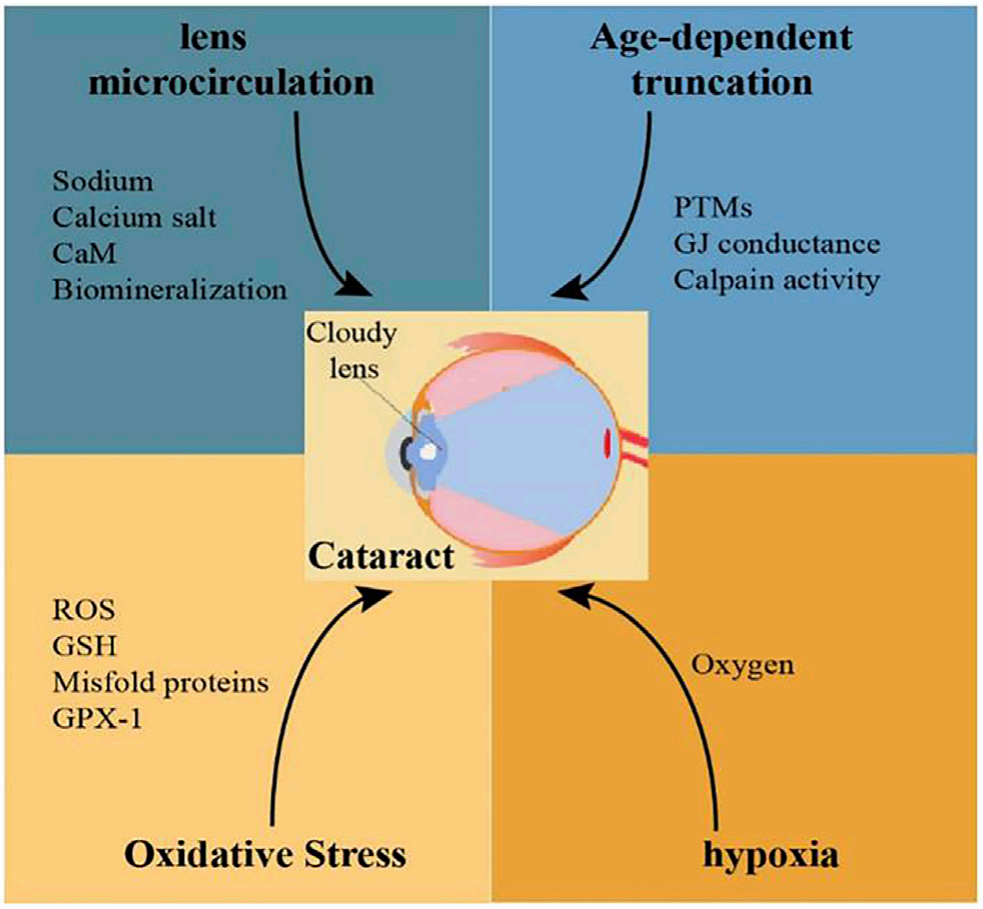
Possible mechanisms related to Cx46 and Cx50 mutations involved in the pathogenesis of cataracts.

Remarkable progress and achievement have been obtained in the last few decades in our basic knowledge of the role of lens connexin hemichannels Cx46 and Cx50 in cataract formation. Connexin variants related to congenital cataracts are being identified in many regions around the world. Adequate and useful animal models have been generated for the investigation of the role of mutant connexin in lens abnormalities during cataractogenesis. The factors that mutate lens connexin in human and rodents and the mechanisms of cataract formation caused by lens connexin mutation and dysfunction could be explored in the future (Figure 2). Despite all the great achievements, much remains to be seen how Cx46 and Cx50 proteins are regulated in the lens under both normal and abnormal conditions. Furthermore, the clinical diagnosis, treatment and prevention based on connexin biology in cataracts are limited. Future investigations should also be arranged to develop effective therapeutic interventions against cataracts.

Mutations of Cx46 and Cx50 in human and rodents can be caused by age, oxidative stress, and hypoxia. Reduced levels of Cx46 and Cx50 proteins or these nonfunctional connexin proteins in lens fiber cells would cause disrupted lens microcirculation, and ultimately, development of cataracts.
